# Tracking of Deformable Objects Using Dynamically and Robustly Updating Pictorial Structures

**DOI:** 10.3390/jimaging6070061

**Published:** 2020-07-02

**Authors:** Connor Charles Ratcliffe, Ognjen Arandjelović

**Affiliations:** School of Computer Science, University of St Andrews, North Haugh, St Andrews KY16 9SX, Fife, Scotland, UK; connor.c.ratcliffe@gmail.com

**Keywords:** computer vision, pose, BBC, articulated, motion, video

## Abstract

The problem posed by complex, articulated or deformable objects has been at the focus of much tracking research for a considerable length of time. However, it remains a major challenge, fraught with numerous difficulties. The increased ubiquity of technology in all realms of our society has made the need for effective solutions all the more urgent. In this article, we describe a novel method which systematically addresses the aforementioned difficulties and in practice outperforms the state of the art. Global spatial flexibility and robustness to deformations are achieved by adopting a pictorial structure based geometric model, and localized appearance changes by a subspace based model of part appearance underlain by a gradient based representation. In addition to one-off learning of both the geometric constraints and part appearances, we introduce a continuing learning framework which implements information discounting i.e., the discarding of historical appearances in favour of the more recent ones. Moreover, as a means of ensuring robustness to transient occlusions (including self-occlusions), we propose a solution for detecting unlikely appearance changes which allows for unreliable data to be rejected. A comprehensive evaluation of the proposed method, the analysis and discussing of findings, and a comparison with several state-of-the-art methods demonstrates the major superiority of our algorithm.

## 1. Introduction

Succinctly put, video tracking concerns the process of determining the location of a moving object as it changes over time. While early tracking research focused on rather specialized domains of application, such as military ones [1], often tracking points using infrared cameras, with the expansion of computer vision, research on the employment of cameras that use visible light has dramatically increased since for two main reasons:

1. Ubiquity
  Video cameras have rapidly increased in availability and image quality, while also decreasing in physical size and cost. Advances in hardware have enabled applications that were previously too expensive, practically cumbersome, or reliant on high fidelity. This includes systems designed for real time and embedded usage.
2. Practical potential
  Video tracking is a cornerstone of computer vision. In order for machines to interact with their environment, they must be able to detect, classify, and track distinct objects much like humans do. Hence, the practical importance of this problem domain is likely to continue increasing artificial intelligence becomes further embedded with everyday life and routine tasks.

The imperative of the aforementioned is easily illustrated by a quick glance at just some of the application domains, the manner in which tracking is employed within them, and the impact that reliable tracking can have. We do this next.

### 1.1. Tracking in Entertainment

Video tracking is widely used in the film industry as a necessary step before combining real imagery with computer generated graphical content (e.g., buildings or crowds of people) that move in coordination with the camera to create a realistic, consistent scene. Another pervasive use of video tracking in films concerns motion capture. Motion capture is the process of translating the pose of a person or an animal to a 3D model corresponding to a structurally similar but possibly imaginary entity, which is then used for animation. Motion capture is typically performed using a great number of physical and easily distinguishable tracking markers (e.g., white dots). This is a laborious and cumbersome process, which is why much of the current work in the field is aimed at achieving marker free tracking. Athletes can be tracked during sports coverage to provide real-time statistics (e.g., the total distance a player has travelled throughout the match) [2]. Interactive systems (e.g., Microsoft Kinect [3]) utilize real-time video tracking to map intuitive gestures to commands [4].

### 1.2. Tracking in Health and Fitness

Video tracking is already widely used to assist medical practitioners. For example, the gait of a patient can be tracked and analysed to instruct rehabilitation or guide instructive intervention. Similarity, athletes’ movement can be tracked to reveal weaknesses and inefficiencies in their technique and direct training efforts [5]. Trackers are also used in precision surgery, allowing for accurate placement and orientation of instruments such needles and bronchoscopes in real time [6].

### 1.3. Tracking in Security

Video tracking is also extensively employed in security applications, often as a pre-processing stage [7,8] to more sophisticated analysis of data. For example, individuals’ movements can be tracked as a means of detecting suspicious and potentially malicious behaviour [9,10]. Video tracking can be used in these situations to draw an observer’s attention to a specific scene, which is particularly useful if one person is tasked with observing multiple screens or areas at once. Pan-tilt-zoom (PTZ) cameras and unmanned aerial vehicles (UAVs) use video tracking to locate people or vehicles (e.g., military convoys or vehicles in high traffic urban areas) [11,12].

### 1.4. Tracking in Scientific Applications

Last but not least, the use of automatic visual tracking is abundant in a wide range of core scientific research. In biology, for example, on the microscopic level, cell tracking is of major interest in different contexts [13,14]. On the macroscopic scale, the tracking of animals (for the study of migration, interaction, or feeding patterns, etc.) is frequent [15]. Particle accelerators (such as synchrotrons) also make extensive use of computer vision tracking, amongst others as a means of studying spatio-temporally varying phenomena, and for accurate sample placement and manipulation [16].

## 2. Previous Work

As noted already, the problem of tracking is pervasive in computer vision and posed in general terms it is a very general challenge, in that it can take on a wide variety of forms depending on the nature of task under the consideration. Hence, a comprehensive review of this extremely broad area of research is well beyond the scope of the present article. Having said that, we would like to start with a general summary of the key considerations that are universally present, both because this helps with the conceptualization of any method as well as because this systematicity is important in understanding where precisely the key novelties and contributions are.

Specifically, the general process of object tracking can be thought of as comprising five stages (though it should be noted that these do not necessarily occur in a clearly delineated form or strictly sequentially). The first four stages are repeated for each video frame processed and concern the actual tracking of objects. The final of extracting useful information from the metadata generated during tracking occurs at the end but is important to include in analysis because it is this ultimate aim of the entire process that contextualizes and gives meaning to what accurate tracking is in a given application. The stages are:

1. Feature extraction
  The data in the given video frame is processed to obtain information useful for detection and tracking. This could involve analysing low-level features [17,18]—such as colour or brightness—to detect edges or interest points. Colour layout descriptors (CLDs) and histograms of oriented gradients (HOGs) [19] are examples of feature detectors. This step may utilize a separate object detector.
2. Object representation
  The shape, appearance, and location of the target are encoded in the target state. The state should balance accuracy with invariance: an accurate model will ignore clutter, reducing false-positives; an invariant model will allow for variable perspectives or illumination levels, reducing false-negatives.
  The shape is usually modelled as a centroid (e.g., a radar response), bounding rectangle (e.g., an approximation of the outline of a vehicle), bounding ellipse (e.g., the outline of a ping pong ball), chain of points/contour (e.g., the outline of a person), or constellation (e.g., the eyes, eyebrows, nose, mouth, and ears of a face) [20].
  If the model’s shape does not exactly fit a given object, then parts of the background will be included, or parts of the object will be excluded. If the model is fixed to a precise shape then it will be inflexible: if the tracked object changes shape, or the video changes perspective, then the model will no longer be representative.
3. Trajectory formation
  The current and previous states of the tracked object are used to predict its future trajectory. This estimate can then be used to help locate the same object in the next video frame. Trajectories can be used to distinguish between neighbouring or occluding objects.
4. Metadata transformation
  The metadata generated during tracking (e.g., the number of objects tracked, and the lifetime of each object) is processed to generate useful information. For example, a tracker used in a retail environment could output the number of customers (i.e., the number of moving objects tracked, excluding employees), and the length of time each customer spends in the store (i.e., the lifetime of each moving object, excluding employees). A CLD could be used to exclude objects that have a similar colour distribution to the employee uniform. It could be assumed that only employees would stand behind the store counters, allowing the tracker to exclude all objects behind the counters.

### 2.1. Overview of Tracking Models

The state of a target comprises its representation and location. A target representation is a model of the object that is being tracked, specifying by such characteristics as the object’s shape and/or appearance. The model can be user-defined prior to tracking, a snapshot of the target, or learnt from training samples (as in the present work). A robust model will continue to be representative of the target despite the presence of extrinsic changes, such as illumination variation or viewpoint drift. It is useful to distinguish between three categories of shape representations: basic, articulated, and deformable.

#### 2.1.1. Basic Shape Bound Models

**Figure 1:**
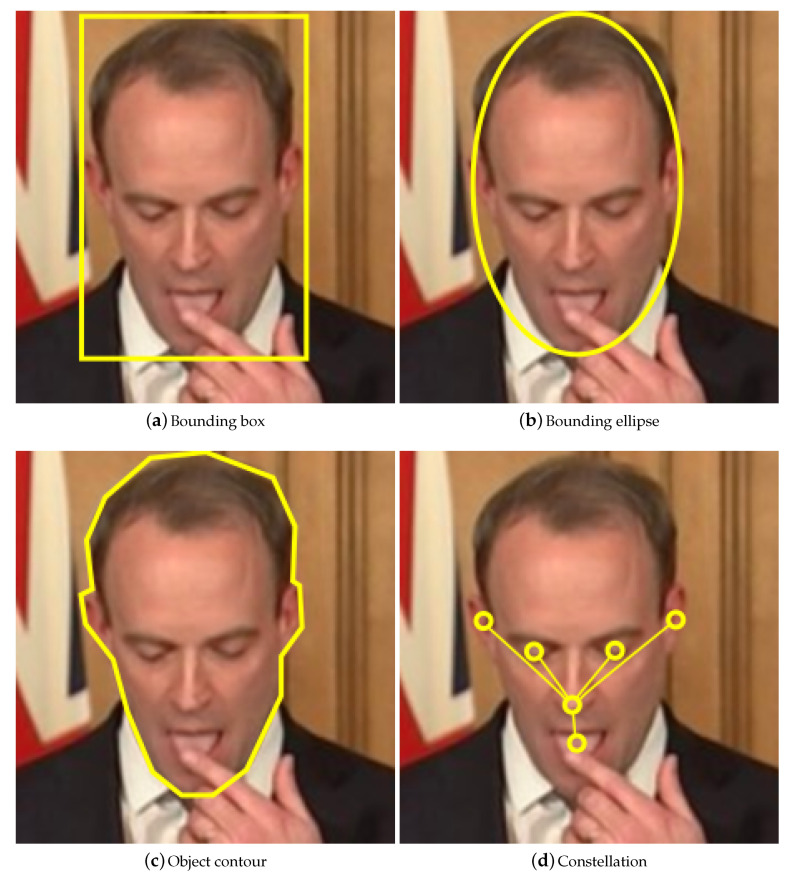
Examples of some common tracking models.

Basic models treat objects as points [21], 2D regions (e.g., rectangles or ellipses), or 3D volumes (e.g., cuboids or spheres), as illustrated in Figure 1a–c. Single point based models inherently do not account for the size of objects, and therefore cannot detect occlusions. Nevertheless, they are frequently used in radar trackers, as the objects being tracked (e.g., submarines or planes) are unlikely to experience occlusion together for extended periods of time.

The appearance of a basic model may be represented by a variety of means, for example by the corresponding colour distribution, simple appearance, or intensity gradient distribution. Neighbouring locations in successive frames are then searched for the best match to this pattern (representation). Using more than one camera can provide multiple views of the same target and enables volume-based models.

The use of more complex shapes (e.g., the precise 3D shape of a car) can reduce the chance of false positive matches by virtue of greater robustness to clutter e.g., by excluding confounding image content that a simple model such as a bounding box would necessarily include. However, complex shapes can be less capable of handling occlusions and changing perspectives, and may increase the probability of rejecting the correct tracking hypothesis i.e., the false negative rate. Trackers relying on specific shapes are also limited in flexibility and inherently specific to a particular domains: an object can only be tracked successfully if there exists a correct shape model in the tracker database of possible shapes.

#### 2.1.2. Articulated Models

An articulated model comprises a set of simpler, more compact basic models related by geometric constraints. Constellations are a typical articulated model (see Figure 1d), composed of a central, key object element, lined to and “surrounded” by other, peripheral elements. A geometric constraint on a constellation may for example force the peripheral elements to stay within a certain distance from the central one. Motion capture trackers model each part separately, forming a constellation of the complete skeleton.

#### 2.1.3. Deformable Models

Deformable models are used when no or little prior knowledge of the target’s characteristics is available, making them widely useful as general object trackers. They are also used when the target’s shape may change in unpredictable ways. A deformable model tracks the individual parts of objects, similarly to articulated models.

While articulated models match the individual components of the target against the corresponding statistical models (e.g., of appearance and shape), deformable models select and then track contiguous interest regions or neighbouring salient points. An interest region could be a large region of homogeneous colour (e.g., a pair of trousers); a salient point could be a small region with a steep intensity gradient. By the very nature of the model, i.e., its deformability, these components are not strongly restricted by motion or distance constraints.

#### 2.1.4. Appearance Models

The precise appearance of a target is usually unique to that specific target instance rather than, say, to a class of targets that it belongs. For example, consider a situation where many people are being tracked. While humans as a class may be considered to have roughly similar shapes due to their shared anatomical structure, there is significant variability due to differing colours of skin, hair, or clothing.

Changing illumination, viewpoint, and clutter, amongst other confounding extrinsic factors also alter the appearance of a tracked object. Models can be updated over time to account for appearance changes. However, this can lead to the model drifting whereby the model gradually becomes less representative of the target’s true appearance due to a gradual inclusion of extrinsic variability. To a degree, this can be ameliorated by incorporating the original model in all incremental updates.

### 2.2. Articulation in Tracking

Considering the focus of the present work and its contributions, we would like to discuss the most relevant work on articulated tracking in some more detail. As the starting point and for the sake of clarity, it is worth emphasising the difference between the tracking of articulated objects and tracking using articulated models. As noted earlier, the former describes models which comprise a set of simpler, more compact basic models (themselves usually rigid) constrained by geometric and kinematic assumptions. In contrast, the tracking of articulated objects may in fact employ non-articulated models [22]—the articulation in this case refers to the nature of the tracked objects itself. Thus, for example, while the human body itself is articulated (limbs move relative to the torso and to each other, but each, say, forearm it itself rigid), the tracking of humans can in many applications be satisfactorily accomplished using non-articulated models such as point clouds [21], silhouettes [23], or deformable templates [24,25].

Having noted that articulated objects can be successfully tracked using non-articulated models, an important benefit conferred through the use of articulated models lies in the explicit recovery of articulation parameters. While not universally this information often is of interest. For example, the articulation of a human hand is useful for hand gesture analysis, of the human body for action recognition [26], and of the spine for time lapse registration of anatomical structures [27]. Indeed, a vast majority of work in the field focuses on the human body [28]. Thus, early and arguably some of the simplest articulated models employed stick figure based models [29,30], which reasonably well suit the shape of humans. However, these fail to generalize, in that they are unable to capture well the shape of a wider range of objects; equally, the absence of any appearance information makes stick models insufficiently robust for most modern applications. Hence, all but without exception more recent articulate tracking approaches employ both appearance and geometric information, and sometimes kinematic constraints as well. Another method of tracking articulated objects using non-articulated models worth mentioning is that of using template banks [31,32]. Rather than employing a generative approach whereby articulation parameters are modelled and recovered implicitly, this approach discriminative in nature—a fixed set of templates corresponding to different articulations is matched against the observed data (video frames). Nevertheless, the vast majority of the work in the area is model based with explicit inclusion of latent variables which capture articulation, and most of these approaches can be seen as falling under the umbrella of pictorial structure based methods. As we will expand upon in further detail in the next section (seeing that the proposed method belongs to this group too), a pictorial structure is a part based model of an object with flexible connections between some of the pairs of parts, encoding and enforcing spatial constraints and statistical relationships between them [33]. Thus, in the influential work of Ramanan et al. [26], the entirety of the human body is modelled as a concatenation of body parts represented by rectangles, the appearance of each of which is modelled separately, and geometric constraints between them. More recent methods extend this idea, employing geometrically more flexible part models [34]. Indeed, numerous variations on the theme have been described in the existing literature, and from this body of work it can be distilled that there are several key aspects which affect tracking performance in different contexts. The main ones concern choices of (i) the extent of object coverage (whether the entirety of an object’s visual appearance is modelled, or only a part thereof), (ii) the manner in which geometric or kinematic constraints are imposed (probabilistic, spring like, etc.), (iv) the representation used to model the appearance of different model parts, (v) if and how model adapts to new information, and (vi) novelty handling (e.g., in the context of occlusion, including self-occlusion). As we explain in the next section, and explore further in the follow-up empirical evaluation, the method proposed in the present paper considers carefully each of these in turn, making greater or lesser contributions, all of which cumulatively give rise to a highly successful general tracker.

## 3. Proposed Method

In this section, we lay out the technical detail underpinning the proposed tracking approach. Considering its nature, we proceed in a top-down fashion, focusing on the pictorial structure design first and then lower and lower constituent elements thereof.

### 3.1. Pictorial Structures Based Modelling

**Figure 2:**
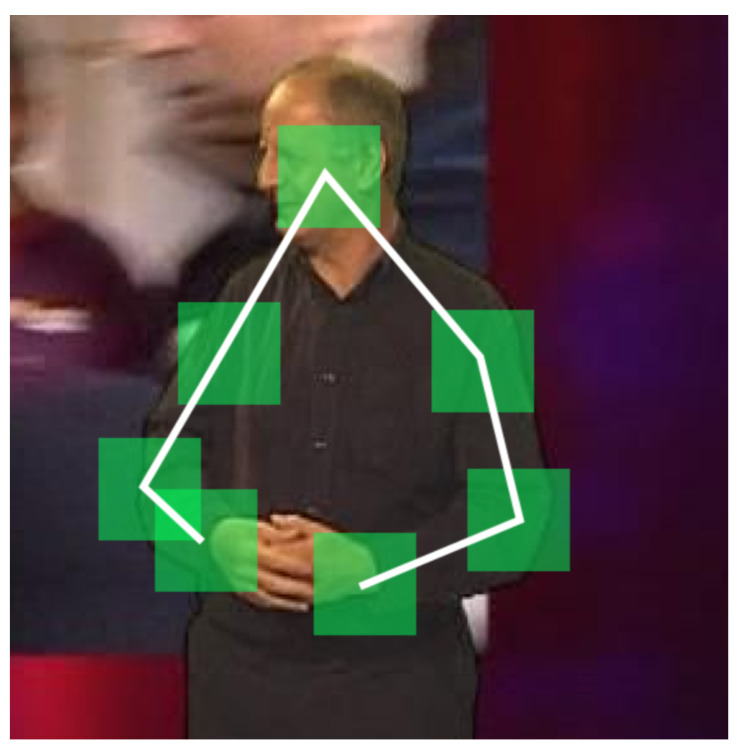
Example of a pictorial structure used to track articulated human upper body motion. The structure comprises seven parts (namely the head, shoulders, elbows, and wrists) and six statistically constraining connections (namely between the head and the shoulders, the shoulders and the elbows, and the elbows and the wrists).

A pictorial structure is a part based model of an object, with flexible connections between some of the pairs of parts (encoding and enforcing spatial constraints and statistical relationships) [33], as illustrated in Figure 2. The appearance of each part is modelled independently of all others, as are the connections between different parts. The general pictorial structure framework is thus highly flexible and does not itself inherently dictate a specific manner for modelling each part or pairwise connection.

As a semantic note included for the sake of avoiding confusion, the kind of tracker we propose here is usually described as a *model free tracker*, even though it uses a model of a kind—namely a pictorial structure. This is because this kind of model itself is only very loosely specified a priori. Instead, the actual model is learnt from the given training data, learning on the fly the appearance and spatial parameters suitable to the specific object being tracked.

The part configuration that is the closest match to a pictorial structure is found by minimising an energy function. This energy function sums the total appearance costs and spatial costs of placing each part, and each pair of parts, in a given configuration.

#### 3.1.1. Part Representation

The role of the global pictorial structure described previously is that of constraining the extent and nature of geometric distortions between different characteristic parts which themselves experience little deformation but can nevertheless exhibit variability in appearance (e.g., due to illumination or viewpoint change). Hence, ultimately, the primary tracking power rests of representing each part in a manner which is robust enough to this variability. Clearly, for best tracking in any particular application, the choice of representation should be domain specific. On the other hand, for a general purpose tracker, the representation has to be able to capture appearance well enough over a wide class of parts, the inevitability being that of sacrifice of best performance in a specific task for good performance across a range of tasks. Hence, motivated by their performance in a wide variety of recognition and tracking problems, herein we adopt the use of the histogram of oriented gradients (HOGs) as a means of representing part appearances [19]. For future reference, we will denote by *F* the dimensionality of HOGs representations.

#### 3.1.2. Feature Extraction and Displacement Estimation

To formalize the tracking setting, our method aims to track *n* distinct parts from frame to frame. For example, in our experiments on the BBC Pose data set [35], n=7 parts are tracked: the head (one), shoulders (two), elbows (two), and wrists (two). The part locations in the first $K_{0}$ frames are used for training. Multivariate Gaussian distributions are used to model the statistical variability in appearance and spatial relationships of the parts. The index of the current frame being used for training or tracking is denoted by the index $f\in Z_{\geq 0}$.

For each frame index $f\geq K_{0}$, the spatial configuration of the parts is extracted and herein denoted by $l^{\left(f\right)}={\left[l_{1}^{\left(f\right)},\;l_{2}^{\left(f\right)},\;\ldots ,\;l_{7}^{\left(f\right)}\right]}^{T}$, where $l_{j}={\left[x_{j}^{\left(f\right)},y_{j}^{\left(f\right)}\right]}^{T}$ denotes the estimated Cartesian frame coordinates of the *j*-th part in the *f*-th frame.

A square patch (a subset of the full RGB image matrix) with a side length of *p* pixels is centred at each part location. The *F*-dimensional image vector of the *j*-th part’s patch in the *f*-th frame, computed using a HOG, is denoted by $i(l_{j}^{\left(f\right)})$.

The displacement vector of the *j*-th and *k*-th parts in the *f*-th frame is:

(1) $$\begin{array}{c} d\left(l_{j}^{\left(f\right)},l_{k}^{\left(f\right)}\right)=\left\{\begin{array}{cc} l_{j}^{\left(f\right)}-l_{k}^{\left(f\right)}, & \;\mathrm{if}\;j<k\\ l_{k}^{\left(f\right)}-l_{j}^{\left(f\right)}, & \;\mathrm{otherwise} \end{array}\right. \end{array}$$

Note that the inequality j<k is required because the spatial correlation of the parts, based on displacement, incorporates both the direction and the distance between parts. Arbitrarily taking the displacement in both directions would give an erroneous spatial correlation.

Prior to training, the ground truth part locations are loaded into an array for constant-time access. The first $K_{0}$ training frames are then iterated over. For each part, the vectorized image patch is computed. For each pair of parts, the displacement vector is computed. An appearance training matrix $A_{j}\in R^{K_{0}\times F}$ is built for each part, and a spatial training matrix $S_{jk}\in R^{K_{0}\times F}$ is built for each pair of parts. The *f*-th row in $A_{j}$ is ${\left(i\left(l^{\left(f\right)}\right)\right)}^{T}$ , while the *f*-th row in $S_{jk}$ is ${\left(d\left(l\left(f\right),l^{\left(f\right)}\right)\right)}^{T}$. These matrices contain the observations used for maximum likelihood estimation.

In order for the HOG to be computed, the frame must first be converted to its corresponding colour matrix. As HOGs are computed multiple times in each frame, a considerable optimisation is obtained by pre-converting each image frame to its colour matrix. These colour matrices are stored in an array for repeated constant time access.

#### 3.1.3. Statistical Model Fitting

Maximum likelihood estimation uses observations to estimate the parameters of a statistical model. Herein, appearance is modelled with an *F*-dimensional Gaussian distribution, and spatial correlation is modelled with a two-dimensional Gaussian distribution. Note that each part (and each pair of parts) is modelled using a different Gaussian distribution, with its corresponding mean and covariance independent of others.

To contextualize the idea, in our case, an appearance observation is an *F*-dimensional representation of an image patch (corresponding to a part image region), while a spatial observation is a two-dimensional displacement. Thus, matrices $A_{j}$ and $S_{jk}$ contain these observations.

#### 3.1.4. Appearance Parameter Estimation

The appearance parameters of each part comprises its mean, $\mu_{j}\in R^{F}$ , and covariance, $\Sigma_{j}\in R^{F\times F}$. Once $A_{j}$ has been constructed, $\mu_{j}$ and $\Sigma_{j}$ can be estimated using maximum likelihood estimation. Thereafter, to ensure robust estimation, the SVD of $\Sigma_{j}$ is computed to give ${\bar{U}}_{j}{\bar{L}}_{j}{\bar{U}}_{j}^{T}$, where ${\bar{U}}_{j},{\bar{L}}_{j}\in R^{F\times F}$. Intuitively, $\Sigma_{j}$ captures the distribution of a part’s appearance in the original HOG space. However, as widely recognized in the literature, only some of this appearance—i.e., a subspace of the original subspace—can be considered reliable, with the remainder of the variation more appropriately treated as noise [36,37]. The dimensionality of this subspace, *m*, inherently depends on the complexity of the appearance variation of a specific part (we analyse this empirically in Section 4. Thus, ${\bar{L}}_{j}$ is then truncated to the *m* greatest eigenvalues by discarding rows from the bottom and columns from the right. ${\bar{U}}_{j}$ is correspondingly truncated to the *m* leftmost columns. These truncated matrices are denoted by $L_{j}\in R^{m\times m}$ and $U_{j}\in R^{F\times m}$, respectively. The inverse covariance is approximated using $\Sigma^{-1}\approx U_{j}L_{j}U_{j}^{T}$.

To account for appearance changes during tracking, $U_{j}$ and $L_{j}$ are used for incremental updating of the covariance in an efficient manner. The matrix $\Sigma_{j}$*itself* is not used in any computations, so it can be discarded [38]. However, $\Sigma_{j}^{-1}$ is used to calculate the squared Mahalanobis distance, and so it is kept. Previous work has explored the possibility of enriching $\Sigma_{j}$ with a latent random variable but found that doing so does not improve tracking significantly. Therefore, herein $\Sigma_{j}$ is used without modification.

#### 3.1.5. Spatial Parameter Estimation

As noted earlier, the statistics of the spatial relationship between of each pair of parts is modelled with a separate multivariate Gaussian distribution. The distribution for the *j*-th and *k*-th parts is captured by its mean, $\mu_{jk}\in R^{2}$, and covariance, $\Sigma_{jk}\in R^{2\times 2}$. Both $\mu_{jk}$ and $\Sigma_{jk}$ are learnt using maximum likelihood estimation, with $S_{jk}$ being the observation matrix.

The spatial correlations can be considered as edges in the complete graph of parts, as shown in Figure 2. The weight $w_{ij}$ of the edge between the *j*-th and *k*-th parts is found by taking the product of the squared Mahalanobis distances of the training displacements:

(2) $$\begin{array}{c} w_{ij}=\prod_{f=0}^{K_{0}-1}{\left(d\left(l_{j}^{\left(f\right)},l_{k}^{\left(f\right)}\right)-\mu_{jk}\right)}^{T}\Sigma_{jk}^{-1}\left(d\left(l_{j}^{\left(f\right)},l_{k}^{\left(f\right)}\right)-\mu_{jk}\right) \end{array}$$

Not all of the spatial correlations are required for tracking—hence by design our method imposes a tree-like structure, with the correlations only between parts which neighbour each other (i.e., which are connected by an edge) in the tree being captured. The weight of each edge is computed, and then the optimal tree is found using Prim’s minimum spanning tree (MST) algorithm.

The aforementioned design facilitates fast parameter computation and efficient model representation. For example, in our experiments on the BBC Pose data set, the node corresponding to the interpreter’s head is chosen as the initial vertex (the 0-th part). When an edge is to be added to the tree, the node currently in the tree stores the new node as its child. The child node stores the mean and covariance for the spatial correlation with its parent. Additionally, each node stores its own appearance parameters. This allows the part configuration to be estimated by performing a tree traversal. Once the MST is complete, the unused spatial correlations (corresponding to edges absent from the MST) can be discarded.

#### 3.1.6. Part Configuration Estimation

At this stage, we are considering the operation of our algorithm when training is completed and the aim is to perform updating tracking of parts in a given video. Herein, we employ recursive configuration estimation (RCE) which is a type of energy minimisation algorithm. While RCE is not guaranteed to produce a globally optimum solution, it has proven to be highly successful in practice and is more efficient than alternatives explored by others [39].

RCE performs a depth-first search of the parts tree introduced earlier. When a node is visited, the corresponding cost function is minimised to estimate the best location for the respective part. The search begins at the initial, root node or part (e.g., in our experiments on the BBC Pose data set, this node corresponds to the interpreter’s head). Its optimum location can be computed using a minimization procedure which can be formally succinctly expressed as follows:

(3) $$\begin{array}{c} l_{0}^{\left(f\right)}=\arg\min_{\bar{l}\in \mathrm{range}\left({\bar{l}}_{0}^{\left(f-1\right)}\right)}a_{0}\left(\bar{l}\right), \end{array}$$

where

(4) $$\begin{array}{c} \mathrm{range}\left(l\right)=\left\{{\left[x,y\right]}^{T}\in R^{2}:|x-x_{l}|,|y-y_{l}|\leq r\right\}\;\mathrm{and}\;l={\left[x_{l},y_{l}\right]}^{T}. \end{array}$$

Once the root location is determined in a frame, the best locations of the roots’s children are found using a related minimization process. In particular, the important difference is that when estimating the location of children, the spatial cost in relation to the parent location is included:

(5) $$\begin{array}{c} l_{j}^{\left(f\right)}=\arg\min_{\bar{l}\in \mathrm{range}\left({\bar{l}}_{j}^{\left(f-1\right)}\right)}\left[a_{j}\left(\bar{l}\right)+s_{jk}\left(\bar{l},l_{k}^{\left(f\right)}\right)\right], \end{array}$$

where the *j*-th part is a child, and the *k*-th part the parent. Note that this minimization relies on the parent’s location in the current frame: our depth-first search ensures that each node is only reached once its parent has been visited. In other words, a part’s location estimation is performed only after its parent’s location estimate is already done.

#### 3.1.7. Incremental Appearance Update

The appearance of each part changes between frames as the interpreter changes pose. In order to account for these changes, the appearance parameters of each part, namely $\mu_{j}$ and *implicitly*$\Sigma_{j}$ (recall from Section 3.1.4 that it is $\Sigma_{j}^{-1}$ which is what our algorithm uses directly and not $\Sigma_{j}$ which is why an estimate thereof is not kept), are incrementally updated.

To be precise, $\mu_{j}\in R^{F}$, $U_{j}\in R^{F\times m}$, $L_{j}\in R^{m\times m}$, and $\Sigma_{j}^{-1}\in R^{F\times F}$ updated in each frame, immediately after the new part configuration has been computed. The new parameters are denoted by ${\hat{\mu }}_{j}$, ${\hat{U}}_{j}$, ${\hat{L}}_{j}$, and ${\hat{\Sigma }}_{j}^{-1}$.

In order to update the appearance parameters of a part, the vectorized image patch (at the new location, in the current frame, computed as described previously) is extracted first: $I_{j}=i\left(l^{\left(f\right)}\right)\in R^{F}$. The component of $I_{j}$ orthogonal to $U_{j}$ is then $G_{j}=I_{j}-U_{j}U_{j}^{T}I_{j}\in R^{F}$.

When *m* is small, the appearance parameters change substantially in each increment. This has the effect of capturing the appearance of a part in recent history (i.e., the most recent frames) and rapidly discounting past appearance information [40]. Equivalently, the appearance parameters model an incomplete representation of the part. To ameliorate this weakness, $G_{j}^{T}I_{j}$ is divided by the *appearance divisor*, $\delta \in R_{\geq 1}$ when constructing $R_{j}$. Herein, we used $\delta =1+{\left(F-m\right)}^{2}$.

Thus, the updating process begins by singular value decomposition of the matrix $R_{j}$

(6) $$\begin{array}{c} R_{j}=\left[\begin{array}{cc} L_{j} & U_{j}^{T}I_{j}\\ 0 & G_{j}^{T}I_{j}\delta^{-1} \end{array}\right]\in R^{\left(m+1)\times (m+1\right)}, \end{array}$$

thus giving $R_{j}={\tilde{U}}_{j}{\tilde{L}}_{j}{\tilde{U}}_{j}^{T}$ where ${\tilde{U}}_{j},{\tilde{L}}_{j}\in R^{\left(m+1)\times (m+1\right)}$.

The new covariance is given by:

(7) $$\begin{array}{c} \Sigma_{j}=X_{j}R_{j}Y_{j}=\left(X_{j}{\tilde{U}}_{j}\right)R_{j}\left({\tilde{U}}_{j}^{T}Y_{j}\right), \end{array}$$

where:

(8) $$\begin{array}{c} X_{j}=\left[U_{j},G_{j}\right]\in R^{F\times (m-1)}, \end{array}$$

thus resulting in ${\hat{U}}_{j}=X_{j}{\tilde{U}}_{j}$ and ${\hat{L}}_{j}={\tilde{L}}_{j}$. Note that ${\hat{U}}_{j}\in R^{F\times (m+1)}$ and ${\hat{L}}_{j}\in R^{\left(m+1)\times (m+1\right)}$, so the bottom row of ${\hat{L}}_{j}$ is discarded, as well as the rightmost column in each of ${\hat{U}}_{j}$ and ${\hat{L}}_{j}$. The new inverse covariance is then simply ${\hat{\Sigma }}_{j}^{-1}={\hat{U}}_{j}{\hat{L}}_{j}{\hat{U}}_{j}^{T}$. Finally, the new mean becomes the weighted mean of $\mu_{j}$ and $I_{j}$ as follows:

(9) $$\begin{array}{c} \mu_{j}=\frac{f\mu_{j}+I_{j}}{f+1} \end{array}$$

##### Achieving Additional Robustness

As we noted previously, a problem inherent in tracking with continuing appearance learning is that of ephemeral occlusions. Here, we introduces a method for detecting these which consequently avoids the incorporation of erroneous information in our part appearance representation, which further distinguishes our contribution from the related ones in the existing literature [39].

In order to detect if a part is occluded, the *distortion increment*, $z_{j}\in R$, is calculated immediately after the new parts configuration is computed, and before any parameter update takes place.

Denote the index of the most recent frame where an appearance parameter update took place by $f_{\mathrm{previous}}$. The distortion increment is computed as follows:

(10) $$\begin{array}{c} z_{j}=a_{j}\left(l_{j}^{\left(f\right)}\right)-a_{j}\left(l_{j}^{\left(f_{\mathrm{previous}}\right)}\right). \end{array}$$

Note that both of these appearance costs are calculated using the appearance parameters from the $f_{\mathrm{previous}}$-th frame. Intuitively speaking, $z_{j}$ quantifies how well $i\left(l_{j}^{\left(f\right)}\right)$ (i.e., the representation of the image patch corresponding to the *j*-th parts’s new location) fits that part’s previous appearance model. The appearance of a part usually changes smoothly with a small viewpoint or illumination changes, with limited appearance variability between successive frames [41]. Therefore, $z_{j}$ ought to be low-valued. If a part is occluded, then its appearance will experience a sudden change, and this will be reflected by $z_{j}$ taking on a large and positive value. The appearance parameters of the *j*-th part are therefore only updated when $z_{j}<t$, where $t\in R_{>0}$ is the occlusion threshold.

For convenience and quick reference, the key variables and model parameters are summarized in Table 1.

## 4. Evaluation

### 4.1. Evaluation Corpus—the BBC Pose Data Set

**Figure 3:**
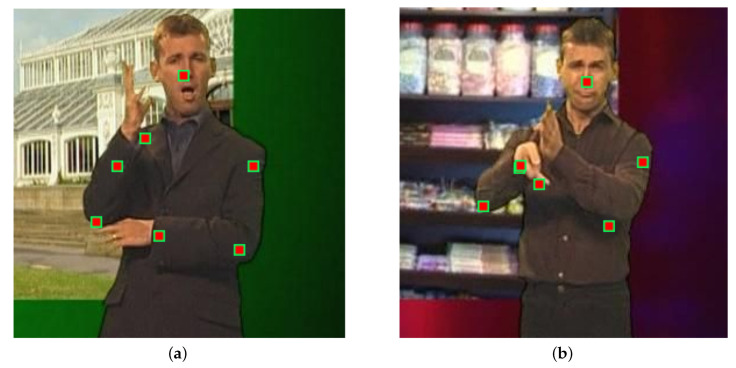
Examples of incorrect labelling in the BBC Pose data set used in our experiments. In (**a**), the marker corresponding to the presenter’s right wrist is misplaced, whereas, in (**b**), the same is the case with presenter’s left elbow as well as the right shoulder.

The BBC Pose data set is well-known and widely used public data set suitable for evaluation of the evaluation of algorithms for tracking articulated objects [35]. It contains a broad range of challenges including occlusions, variable and dynamic backgrounds, and rapid movement of objects (or parts of objects) of interest. However, it should be noted that the nominal ground truth labelling is not error-free, as illustrated in Figure 3. Therefore, any results or comparative analysis using this data set should be caveated by noting that the actual ground truth is not known—rather, what is available is *quasi-ground truth*. Indeed, as we will shortly show, in some instances, our algorithm produces *better* localization of parts than the provided quasi-ground truth.

### 4.2. Results and Discussion

We started out evaluation by looking at the overall performance of the proposed method and its comparison with the existing, state-of-the-art-tracker in the literature. As noted in the previous section, BBC Pose data set is used extensively, which facilitated a straightforward, like for like comparison.

**Figure 4:**
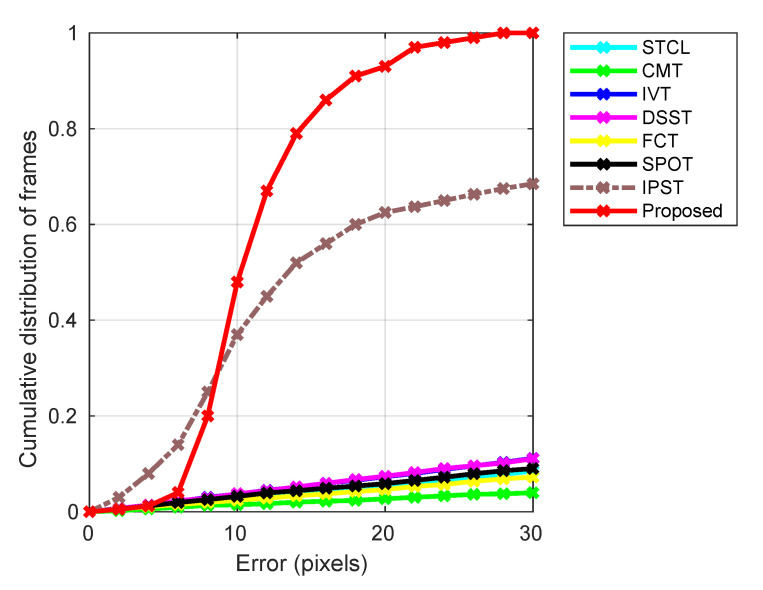
Empirical performance on the BBC Pose data set of six different state-of-the-art methods and the proposed method (see the legend above the plot). The abscissa (i.e., the *x*-axis) shows the average pixel error in the location of different model parts, whereas the ordinate (i.e., the *y*-axis) shows a cumulative distribution of frames corresponding to a specific error. The vastly superior performance of our solution is readily apparent.

The key results are summarized in Figure 4. The plot characterizes the empirical performance (taken as reported in the literature) on the BBC Pose data set of eight different methods (see the legend above the plot), namely the Spatio-Temporal Context Learning (STCL) tracker [42], the Structure-Preserving Object Tracker (SPOT) [24], the Incremental Visual Tracker (IVT) [38], the Fast Compressive Tracker (FCT) [43], the Discriminative Fast Scale Search (DSST) tracker [44], the Clustering Model Tracker (CMT) [25], and the Incremental Pictorial Structure Tracker (IPST) [39] as well as the proposed method (our experiments). Table 2 lists the values of our algorithm’s parameters. These were chosen as sensible values based on our understanding of the application domain. In practice, in cases when no similar information is available, the parameter values can be learned using the standard training-validation-test protocol.

In the plot shown in Figure 4, the abscissa (i.e., the *x*-axis) corresponds to the average pixel error in the location of different model parts, whereas the ordinate (i.e., the *y*-axis) the value of the cumulative distribution of frames corresponding to a specific error. The superior performance of our solution and IPST that is the two pictorial structures based methods is readily apparent. All of the other trackers exhibit similar performance (though with a clear and consistent ranking amongst them) across different error magnitudes with roughly linear dependency of the cumulative proportion of frames. In contrast, both our method and IPST show markedly better behaviour for small errors which gets particularly rapidly distinct up to the error magnitude of 15 pixels, thereafter maintaining a roughly constant (or somewhat increasing) margin of improvement. The difference between the two pictorial structures based methods is interesting and insightful. In particular, in the region of the plot corresponding to small tracking errors (up to 8 pixels), note that IPST tracks in more frames with a very small error, thus exhibiting somewhat better performance than our method on approximately quarter of the frames. However, thereafter, the situation reverses with our algorithm managing to track much better in the majority of the frames. For example, while IPST tracks only about half of the frames with an error smaller than about 13 pixels, our method manages the same with nearly three quarters. The likely reasons for this interesting behaviour stem from several key differences between the two approaches. The most important of these concerns the updating of parts’ appearances, namely our discounting of historic information and rejection of likely occlusions. These make our algorithm more “reluctant” (in a manner of speaking) to the inclusion of new information. Thus, in the presence of appearance changes, the approach is more prone to small, transient errors, which are eliminated once sufficient evidence for novelty is accumulated. Equally, it is exactly the same mechanism that prevents large errors from being introduced by transient occlusion—temporary occluding (mis)information is never incorporated in the model, limiting tracking error both in magnitude and duration. The difference in the manner that the search for optimal parts configuration is estimated is also likely a contributing factor. When conditions are challenging (changing illumination, viewpoint, occlusion), our computationally more efficient search is good enough in the sense that, in this context, it is the other factors that are the greatest source of error. On the other hand, when appearance change is limited, the slower but more exhaustive algorithm used in IPST is better at finding the actual global optimum, absolutely minimizing the invariably error. This observation provides potentially useful insight for future research in that it opens the possibility of choosing on the fly which of the two search algorithms should be employed at a specific point in tracking.

#### Parameter Sensitivity Analysis

**Figure 5:**
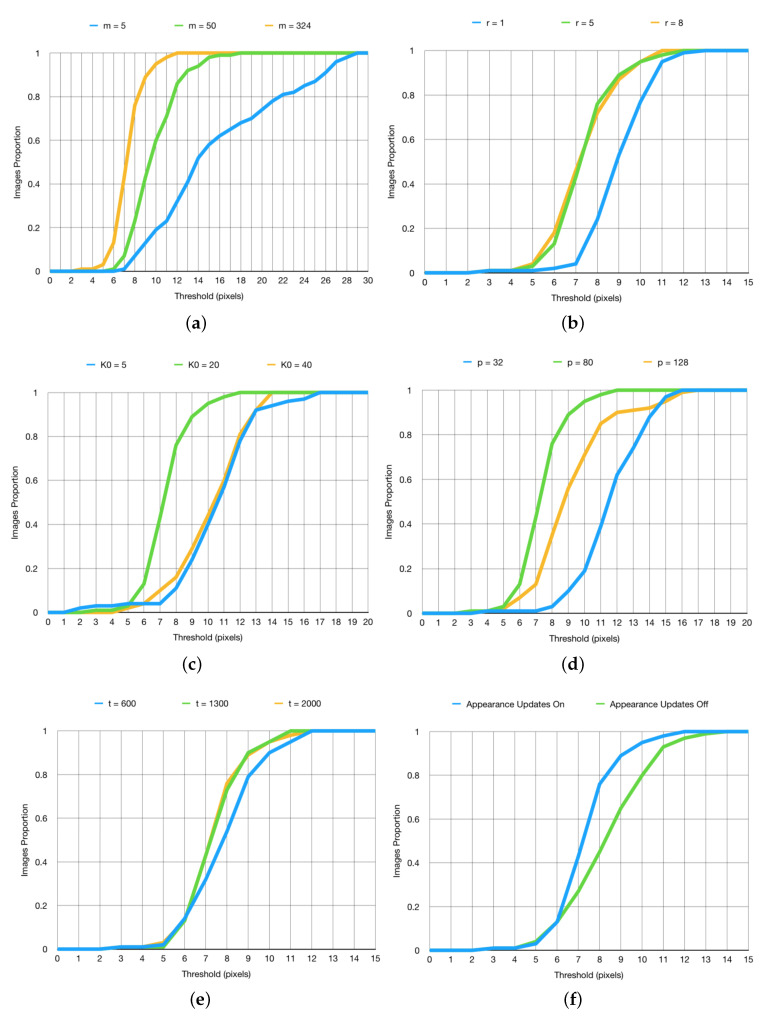
(**a**–**f**) Summary of parameter sensitivity assessment. The *x*-axis values are thresholds for the mean Euclidean distance (measured in pixels) of each part’s tracked location from the ground truth. The *y*-axis shows the proportion of frames in which the mean distance is within the threshold. For example, when m=50 (top-left), the mean distance is less than 10 pixels in 60% of the frames.

Following our initial experiment which demonstrated our method’s vastly superior performance in comparison with the state of the art, in our next set of experiments, we sought to gain further insight into the sensitivity of the algorithm’s performance to the values of its parameters. Our findings are summarized in Figure 5.

As expected, increasing the value of the parameter *m* produced more accurate tracking results, as demonstrated by the plot in Figure 5a. Recall that *m* determines the dimensionality of the subspace used to capture each part’s appearance [37]. Consequently, our finding can be seen to make intuitively sense: a higher value of *m* leads to a more complex and expressive model appearance model, with less information being discarded. This of course comes at a cost. Increasing *m* increases the size of matrices $U_{j}$ and $L_{j}$, thereby imposing greater storage requirements and increasing the time needed to calculate $\Sigma_{j}^{-1}$. Thus, in practice, a balancing act needs performed, with the exact trade-off being highly dependent on the nature of the objects being tracked as well as the environmental context. For example, in Figure 5a, the usual phenomenon of diminishing returns is clear, the change from m=5 to m=50 effecting greater performance differential than from m=50 to m=324.

The effects associated with varying the parameter *r* are similar, both phenomenologically and conceptually. As demonstrated by the plot in Figure 5b, increasing its value improves tracking. Recalling that *r* governs the breadth of the spatial search region in the search for the optimal part location in a frame, the reasons behind this observation are clear: excessively small *r* limits the maximum speed of motion too much, thus not allowing for the correct localization to take place when this speed is exceeded (e.g., in our experiments, this most notably applied to the interpreter’s wrists/hands). Similarly, in line with expectations, increasing *r* past a certain point fails to effect further improvement—this happens when the maximum search window is in excess of the highest rates of movement of tracked parts. Hence, much like in the case of the previous parameter, the optimal value is application dependent. In some cases, depending on the type of objects tracked, the speed at which different parts move is greater and in others lesser. An overly high value of *r* imposes an unnecessary computational cost during the search, whereas an overly low value fails to track correctly.

The behaviour of our algorithm for different values of $K_{0}$, i.e., the number of initial frames used for training, is more interesting; see Figure 5c. Increasing the number of training frames from a small number results in more accurate tracking, as expected (in our experiments up to $K_{0}=20$). Clearly, using too little training data leads to overly simplistic initial model and this oversimplification propagates through time because it limits the ability of the model to adapt appropriately as new information is encountered. However, as the number of training frames is increased past a certain point, contrary to what one may expect at first, a *decrease* in tracking the accuracy is observed. Though this may seen counter-intuitive, it is in fact something that the theoretical underpinnings of our method predict well. In particular, during training, all frames—that is, all the information on parts’ appearance variation—are used on par with one another, not taking advantage of our information discounting (purposeful `forgetting’ of overly old data) which takes place during steady state tracking i.e., the incremental updating stage. As a practical note, as was the case with the previously considered parameters, it should be born in mind that the optimal value of $K_{0}$ is application dependent.

The size *p* of the image patch treated corresponding to a part in our pictorial structure is clearly an important parameter, see Figure 5d. The smaller the size, the more local the corresponding part appearance model is and, conversely, the greater the size, the greater its spatial extent is. Here, we face another, well-known balancing act. The use of smaller, more localized image regions for the representation of parts has an important advantage of being less susceptible to the inclusion of confounding, spatially proximal (in image plane) information (for example from the background). It also inherently has a more constrained appearance which allows for easier and more accurate representation of the corresponding variability. These advantages carry a fundamental cost. Notably, the more constrained appearance and thus lower information content can lead to increased difficulty in precise localization of a part between successive frames. On the other hand, less local, larger image patches make the corresponding modelling of possible appearance changes more difficult, thus requiring more data and more complex models if unwanted appearances are not to be captured too (for example, as may be the case with linear subspace models [45,46]). Equally, there is a greater chance that surrounding, irrelevant information is incorporated in the model. However, the increased information content can lead to better localization performance. As before, the trade-off is invariably application domain specific; for example, in our experiments, we found that the patch size of p=80 offered a suitable compromise between the two extremes and resulting in best tracking, as demonstrated by the plot in Figure 5d.

**Figure 6:**
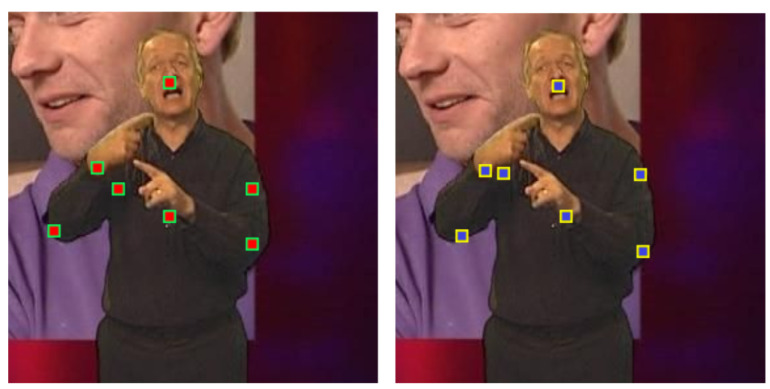
Examples illustrating our observation that in some cases our tracker (right-hand frame) produced *better* estimates of parts’ locations than the quasi-ground truth (left-hand frame) provided with the BBC Pose data set (see Figure 3). This phenomenon can be attributed to the intelligent continuing learning strategy we employ, and its ability to reject unreliable measurements, allowing the method to make sensible choices in periods of transient self-occlusion. In this particular instance, observe the localization of the presenter’s right shoulder.

Lastly, the impact of continuing appearance learning i.e., incremental model adaptation and the corresponding robustness parameter *t* are summarized by the plots in Figure 5e,f. At this point, it is worthwhile reminding the reader of our note as regards the quality of ground truth, that is, what we described as quasi-ground truth, provided with the BBC Pose data set. In particular, we observed that, in some cases, our tracker produced *better* estimates of parts’ locations than the quasi-ground truth. Examples are shown in Figure 6. This can be attributed precisely to the intelligent continuing learning strategy we employ, and its ability to reject unreliable measurements, allowing the method to make sensible choices in periods of transient self-occlusion.

## 5. Summary and Conclusions

In this article, we proposed a novel method which systematically addresses the well-known difficulties in tracking complex, articulated or deformable objects—a highly relevant problem in an era of ubiquitous technology in all realms of our life. On the global label, spatial flexibility and robustness to deformations are achieved by its pictorial structure based geometric model. Appearance description is localized to regions which exhibit little deformation (pictorial structure’s parts) and whose breadth of appearance variability is captured by a linear subspace model in a histogram of oriented gradients space. What makes the proposed method particularly successful is its continuing learning framework of both the geometric constraints and part appearances, which builds upon the usual one-off learning taking place at the onset of tracking. The continuing is achieved through incremental updates of parts’ models that is the corresponding subspace representations, and is made particularly robust through the implementation of occlusion detection and information discounting i.e., the discarding of historical appearances in favour of the more recent ones. Lastly, we described a set of comprehensive empirical experiments in which we compared the proposed method with several state-of-the-art methods in the literature and demonstrated its advantages.

## Figures and Tables

**Table 1:** Summary of key variables underlying the functioning of the proposed algorithm. For full details, please see the main text.

Symbol	Summary Explanation
$l_{i}^{f}$	Cartesian frame coordinates of the *j*-th part in the *f*-th frame.
*p*	Width in pixels of the square image patch used for the representation of parts’ appearances.
$\mu_{j}$	Mean of the *j*-th part’s appearance (HOGs space).
$\Sigma_{j}$	Robust covariance of *j*-th part’s appearance (HOGs space).
*m*	Dimensionality of the linear subspace representing the *j*-th part’s appearance (HOGs space)
$w_{i,j}$	Weight the connection between the *j*-th and *k*-th parts in the parts tree.
*r*	Maximum frame to frame displacement of a part in pixels.
δ	Appearance divisor, used to control the rate of discounting of historical data.
*z*	Distortion increment, quantifying the novelty in a part’s appearance observed in the current frame.
*t*	Occlusion threshold, used to control the extent of permissible novelty in appearance updates (see *z* above).

**Table 2:** The values of our algorithm’s parameters used in our first set of experiments (also see Figure 4).

Parameter	*F*	*m*	*r*	$K_{0}$	*p*	*t*
Value	324	324	5	20	80	2000
